# Antibiotics and hexagonal order in the bacterial outer membrane

**DOI:** 10.1038/s41467-023-40275-0

**Published:** 2023-08-09

**Authors:** Georgina Benn, Thomas J. Silhavy, Colin Kleanthous, Bart W. Hoogenboom

**Affiliations:** 1 Department of Molecular Biology, Princeton University, Princeton, NJ 08544 USA; 2 Department of Biochemistry, University of Oxford, Oxford, OX1 3QU UK; 3 London Centre for Nanotechnology, University College London, London, WC1H 0AH UK; 4 Department of Physics and Astronomy, University College London, London, WC1E 6BT UK

**Keywords:** Antibiotics, Membrane structure and assembly, Cellular microbiology, Nanoscale biophysics

**arising from** S. Manioglu et al. *Nature Communications* 10.1038/s41467-022-33838-0 (2022)

**Fig. 1:**
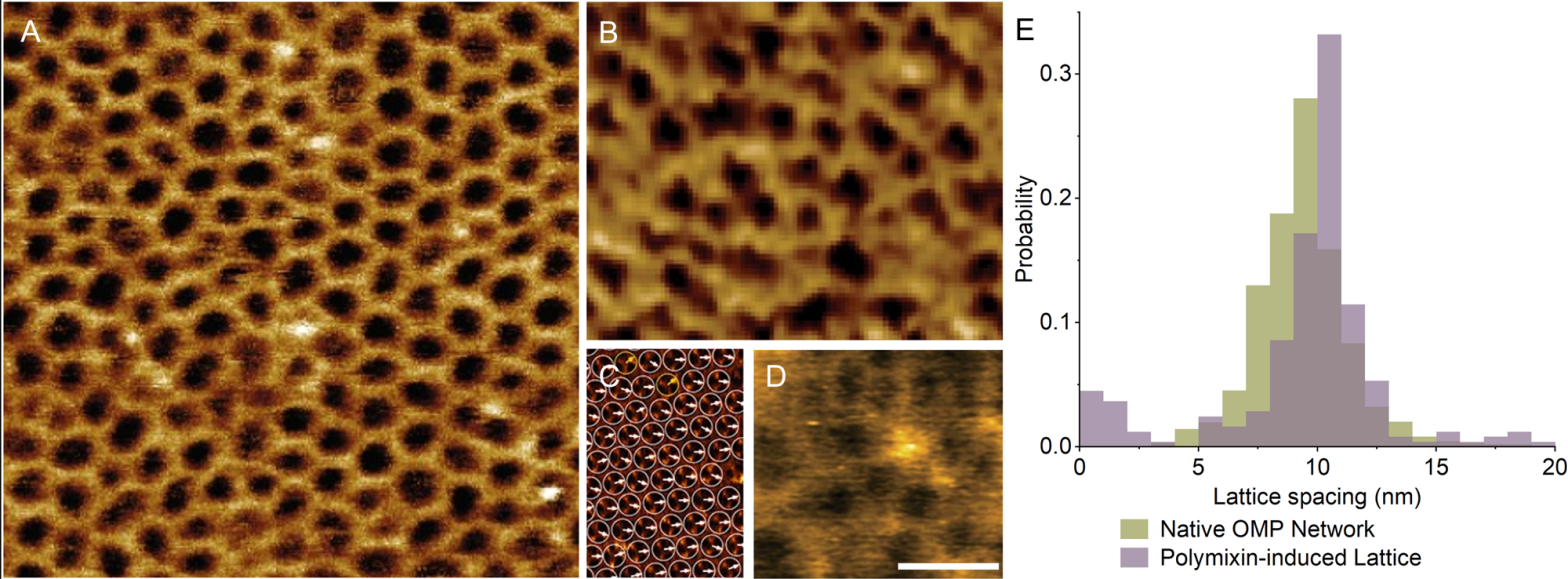
Comparison of ordered configurations in the outer membrane, as imaged by AFM. **A** When polymyxin was applied to purified outer membranes from MG1655 *E. coli*, ref. ^1^ saw a hexagonal lattice with a spacing of 9.8 ± 3.9 nm. **B** Benn et al. ^7^ showed that the native, untreated outer membrane of living MG1655 *E. coli* was dominated by trimeric OMPs arranged in an imperfect hexagonal lattice, with a nearest-neighbour distance of 9.4 ± 2.1 nm. **C** Jaroslawski et al. ^4^ observed hexagonal arrangements of trimeric porins with a lattice constant of 8.1 ± 0.3 nm in purified outer membranes from *R. Denitrificans*. **D** Oestreicher et al. ^6^ showed hexagonal lattices (spacing 10.5 ± 1.7 nm) on the surface of living *E. coli*, which they attributed to trimeric OMPs. **E** Comparison of lattice-spacing distributions for polymyxin-induced hexagons^1^ and for OMP lattices in native (untreated) MG1655 *E. coli*^7^, showing no significant difference by a two-sided t test with unequal variance (*p* = 0.16). Values are mean ± SD. Scale bar is 20 nm. Images A-D were reproduced from references as indicated, with permission.

Polymyxin is a last-resort antibiotic that targets Gram-negative bacteria. It does so by binding lipopolysaccharide (LPS) in the outer leaflet of the outer membrane by an ill-defined mechanism. Recently, ref. ^1^ used atomic force microscopy (AFM) to image outer membrane vesicles ruptured and flattened onto mica in the presence of cations. They then added polymyxins, which resulted in the appearance of a hexagonal lattice in the membrane (Fig. 1A). They attributed this lattice to LPS-polymyxin crystallisation and suggested that such lattices are relevant for the mechanism of action by which polymyxins induce bacterial cell death.

Previous electron microscopy and AFM studies have shown that trimeric outer membrane proteins (OMPs) also pack together in hexagonal lattices, as observed in reconstituted 2D arrays^2,3^, in purified outer membranes^4^ and in living bacteria^5–7^ (Fig. 1B–D). These OMP arrays have lattice constants virtually identical to those seen for the polymyxin-induced hexagons reported by Manioglu et al. (Fig. 1E).

Despite this similarity, Manioglu et al. proposed that polymyxin arranges LPS into such hexagonal arrays independent of OMP content of the membrane^1^. To substantiate this hypothesis, they showed that polymyxin still formed hexagonal arrays in patches derived from outer membrane vesicles from BL21 (DE3) *omp8 E. coli* cells that did not express the primary trimeric porins OmpF, OmpC, and LamB, or the monomeric OmpA, but were enriched in other OMPs^8^. However, we now know that many OMPs^9^, not just trimeric porins, have a propensity to form heterogenous clusters in the presence of LPS. More extensive negative controls would be needed to dismiss OMPs as the basis for the hexagonal lattices that are observed following polymyxin treatment of outer membrane vesicles.

Conversely, the polymyxin-induced lattice was affected by LPS length and cation concentration, from which Manioglu et al. concluded that the lattice was determined only by polymyxin–LPS complexes^1^, aided by the tendency of LPS molecules to form crystalline domains. They cite LPS crystallinity seen in molecular dynamics and in model membranes^10–12^, to suggest that the polymyxin-induced hexagonal geometry is related to LPS packing. However, the hexagonal LPS arrays referenced have lattice constants of approximately one order of magnitude smaller^11^ than the arrays seen by ref. ^1^.

Based on their observations, Manioglu et al. conclude with the suggestion that “a local ordering of LPS by polymyxin must lie at the core of the [hexagonal] arrangement”^1^. In contrast, based on the observations above, we propose an alternative conclusion, assigning the observed hexagonal order to the symmetry of OMP networks in the untreated *E. coli* membrane^7^. The OMP–OMP interactions in such networks are mediated by LPS^9^, which is thereby expected to follow similar local ordering. Hence in our interpretation, the polymyxin does not order the LPS, but binds to the already ordered LPS and thereby reveals existing OMP lattices at a higher contrast than is the case for the untreated membranes.
